# Power-system protection device with IoT-based support for integration in smart environments

**DOI:** 10.1371/journal.pone.0208168

**Published:** 2018-12-05

**Authors:** Octavian Mihai Machidon, Cornel Stanca, Petre Ogrutan, Carmen Gerigan, Lia Aciu

**Affiliations:** 1 Electronics and Computers, Transilvania University of Brasov, Brasov, Romania; 2 Electrical Engineering and Applied Physics, Transilvania University of Brasov, Brasov, Romania; Basque Center for Materials, Applications and Nanostructures, PORTUGAL

## Abstract

This paper proposes a power-system protection device designed to be integrated in smart environments based on Internet-of-Things technologies. The proposed system enhances electrical safety by fast disconnection of the power supply in case of fault events like leakage current, electrical arc, overcurrent or overvoltage and has been designed with the goal to be integrated in smart environments like smart homes or smart cities for protecting the electrical equipment. The system also enables real-time monitoring and notification events through an advanced communication interface using a data concentrator architecture. This paper provides an extended description of the proposed system’s design and implementation, as well as the experimental validation results.

## Introduction

The security and reliability of the electrical energy infrastructure is of vital importance today more than ever, given the degree to which electric-powered technology has become embedded in all human activities. Protecting the electrical power supply system against interruptions due to various faults is thus a main research concern [1]. One of the components involved in power-system protection is the circuit breaker, which is responsible for closing the system when a fault or anomaly occurs in order to protect the electrical equipment [2].

In today’s world, the technological trend of implementing “smart” technologies, fostered by the emergence of Cloud Computing and the Internet of Things (IoT), led to a transfiguration of ordinary devices and environments to “smart” entities [3]. In this context, traditional electrical protection devices also tend to transcend and become “smart” [4], and consequently offer improved fault-detection and protection, remote monitoring and event notification [5].

By becoming smart, a home is embedded with ubiquitous computing equipment that connects all the household devices to one another and the Internet. A smart city also embeds in the urban landscape computers, sensors, cameras and other sensitive equipment operating in the background. In these circumstances, protecting the power supply grid against faults becomes even more important, given the increasing number of sensitive devices connected in the emerging Smart world.

Moreover, the traditional electrical protection for residential, office and industrial buildings is based on classic circuit breakers tripping or fuses being blown when an overload happens, thus offering limited protection and warning [6].

This paper describes the design, implementation and experimental validation of the ELSA (ELectrical SAfety) power-system protection device with built-in support for IoT-based integration in smart environments like a Smart City or a Smart Home. The main features of the proposed system are:

- Advanced fault-detection and protection through high-speed disconnection in the case of: overcurrent, overvoltage, leakage current and electric arc;
- Real-time monitoring by sending recorded events to a Web server where the information is accessible through an online Web-based interface;
- Real-time notifications by e-mail and text messages to designated persons;
- Flexible and scalable communication infrastructure supporting easy integration in smart environments and with other utility service operators through Web-based protocols, services and APIs;

The rest of the paper is organized as follows: section II provides background and related work on this topic, section III describes the system architecture detailing the three components (hardware, software and communication network and data concentrator), section IV presents the experimental validation of the system while section V draws conclusions and underlines future research directions.

## Background and related work

Relevant literature in this domain underlines three key features for such power system protection devices: disconnection speed, reliability and cost [7].

The current state-of-the-art in this field highlights that the traditional circuit breakers are still widely used today for power-system protection, these devices being usually designed to protect against a single power supply grid fault event. A new generation of circuit breakers has been developed by Eaton in 2016: Arc Fault Detection Device (AFDD) which provides enhanced protection against electric arcs, leakage current, overcurrent and overvoltage [8].

One of the recent trends is linked to the emergence of micro-grids, which need to operate safely in both islanded and grid-connected modes. Hence, the re-shaping of the traditional power grid (transcending into Smart Grid and micro-grids) will require highly flexible distribution management systems and a re-design of protection strategies [9]. The reliability of micro-grids is an important research direction, given their sensitivity to power outages which can increase the average frequency and duration of interruptions [10]. Thus, existing research focuses on designing reliable protection solution for such systems [11] (e.g., the authors in [12] provide a comparison between protection solutions for AC and DC micro-grids, highlighting the advantages in the case of DC protection).

The DC micro grid systems are present in an increasing number of applications lately due to their important assets. Thus, protecting DC micro grids and the connected consumers to such grids is an important research subject, addressed by several recent papers. As such, in [13] the authors analyze the multi-terminal protection in DC grids for aerospace applications. In [14] a pilot project for electric vehicle charging stations fed by renewable: PV and train regenerative braking is presented, with details regarding control and protection features. An advanced protection method against voltage anomalies in DC grids is described in [15], where the protection circuit located in the energy converter ensures a fast decoupling and fault identification in a multi-terminal grid. A similar protection system intended for a multi-terminal DC compact node feeding electric vehicles on electric railway systems, secondary distribution networks, and PV systems is also presented in [16].

Another sensitive issue with regard to power system fault-protection is related to the complexity of integrating renewable energy sources in the existing power grid, which underlines even more the necessity of viable solutions against faults like short-circuits, electric arcs, and increased harmonics [17]. Consequently, this highlights the importance of disconnecting power when detecting current zero-crossing. Nevertheless, the assimilation of distributed sources in to existing power distribution networks raises important technical challenges, including issues related to the need of enhanced protection systems against power failures [18].

In this context, the emerging research trend in this field envisages designing and implementing innovative protection solutions, like the one described in a recent patent application [19], which proposes a similar device for ensuring electrical load disconnection when detecting various faults in the power supply grid.

The analysis of the current state-of-the-art shows that there are very few smart circuit breaker—type solutions, and the traditional protection systems do not offer real-time monitoring and communication features for fast IoT-based integration in smart environments. There is an emerging Electrical Substation Communications Standard (IEC-61850) [20] that is Smart Grid compatible and enables communication over the power grid for monitoring and control, however such systems are generally dedicated for power utility providers and specialists, not being designed to provide monitoring or notifications to end-users or third-parties (e.g local public administration entities or emergency services).

Related research papers describing smart circuit breakers (circuit breakers that are remotely controllable and monitored) include smart breakers used for local power generator capacity optimization [21], aircraft electrical power distribution protection [22] and also applications in micro-grids for enhanced grid protection [23] and also optimal load scheduling [24]. A similar but less complex system with the one described by this paper is presented in [25], and it represents a remotely controllable circuit breaker protecting against short-circuits and overloads.

Most of the above-mentioned examples of smart circuit breakers offer the traditional protection features of a classic breaker but in addition provide remote control for coupling/decoupling and monitoring. A recent study [26] analyzed the security implications of having remotely controlled circuit breakers and underlined the necessity of a secure communication channel to avoid possible attacks.

The ELSA power-system protection device described in this paper has been designed with the goal to be integrated in smart environments like smart homes or smart cities for protecting the electrical equipment from faults and anomalies. It not only allows remote monitoring and control of the coupling/decoupling feature, but also supports real-time notifications and provides enhanced protection against a wide range of electrical failures.

The concept of smart buildings, smart homes or home automation has been developed with the goal to make the living environment safer, more convenient and more accessible for the inhabitants [27]. The features that a smart home offers include, among other, environment monitoring [28], building security [29] or remote medical monitoring [30]. Given that a smart home integrates a complex network of devices, sensors and computers, and since this concept also aims at improving living safety, one of the key issues that need to be addressed is related to the monitoring and protection of the power system. In other words, if the equipment inside the home became smart, it is natural to expect that the circuit breaker system should transcend into an intelligent, enhanced protection system.

Given the position of the circuit breaker in a smart building, adjacent to the power meter, we envision our protection system to integrate in the Smart Grid—Smart Home synergy as a system offering active protection but in the same time providing real-time monitoring of the link between the two infrastructures, fostering the secure interaction between the two entities [31].

With regard to the second application domain targeted for our device, smart cities, this was identified based on the collaboration between Transilvania University of Brasov and the local Brasov authorities in the framework of the Brasov Smart City roadmap. Brasov is one of the Central and East-European cities with a strategy for implementing smart ICT-based technologies like intelligent public lighting [32], GIS (Geographical Information System) and video-enabled traffic surveillance for real-time analysis and optimization or WoT (Web of Things)-enabled services like open Smart City Data (e.g. precipitation measurements) [33].

The intelligent public lighting network integrates a remote lighting management system able to monitor all the lights in the city remotely, using a Web-based interface. Also, this system has included the implementation of environmental sensors, video cameras, panic buttons and a municipality-wide Wi-Fi MESH network, all being monitored and managed using the same interface that allows analysis and planning instruments for reducing energy consumption and maintenance costs [34].

In this context, an important issue regards fault identification and equipment protection, which can be addressed properly with the proposed system. Thus, the optical elements of the lighting devices, but also the other equipment connected to this intelligent network (cameras, microphones, alarm systems, sensors) can be protected against a wide range of power system faults which are identified and signaled in real-time.

The public lighting poles part of the IoT-based intelligent lighting system integrate only a basic circuit breaker offering decoupling in the case of a short circuit. Thus, the ELSA device provides additional protection against a wider range of electrical faults. This is also the case of residential or industrial buildings, most of which are protected only against short-circuit (and only some having differential breakers). In both cases, deploying the ELSA device enables, besides enhanced protection, real time notification of the public energy provider or the building owner about the fault events as they occur.

## Materials and methods

### Hardware implementation

#### General description

**Fig 1:**
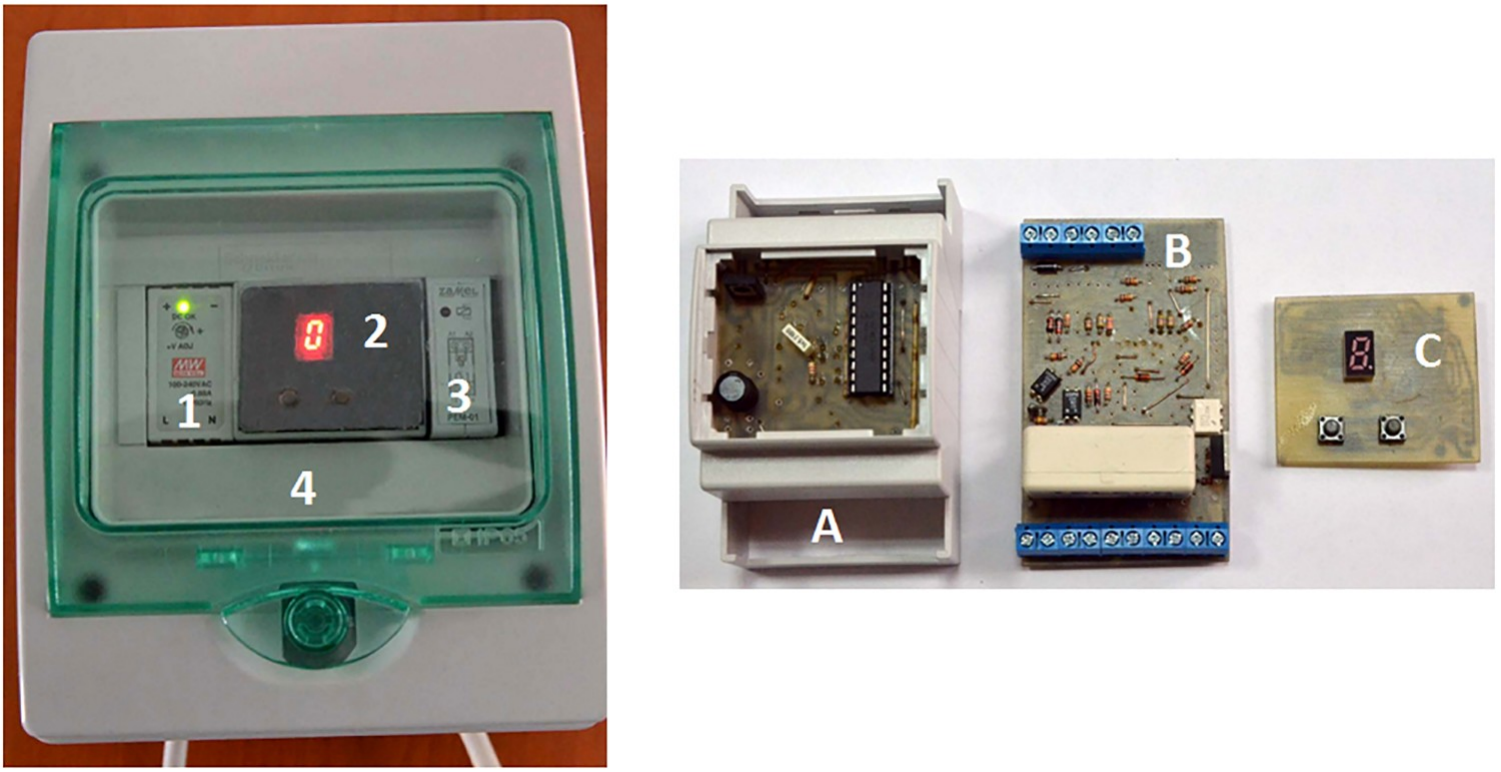
The power system protection device.

The ELSA protection device described in this paper (and illustrated in Fig 1) has been implemented based on an original, innovative design, and ensures the protection of electrical consumers connected to the public power supply grid by disconnecting the electrical supply in the event of several faults: overvoltage, overcurrent, leakage current and electrical arc. In the left subfigure the ELSA device is displayed in its case, with the modules being identified as follows:

1. Power supply (230V AC to 12V DC)
2. Control module containing three stacked PCBs (A, B, C from the right subfigure)
3. Relay (SW2) for neutral switching
4. Casing including the solid state relay (SW1)

The right subfigure shows the microcontroller unit with its three overlayed PCBs:

(A) PCB for microntroller and switched supply sources
(B) PCB for sensors
(C) PCB for user control (via the buttons) and states display

The main features of the protection system are:

- All the protection sub-systems are integrated in a single device, mounted in a DIN Rail enclosure to ensure flexibility in choosing the appropriate protection modules for each application (As seen in Fig 1).
- Rapid fault detection—in under 250us.
- Fast electrical load decoupling (maximum 10ms) which takes place at the first current zero-crossing following the fault event for overvoltage, leakage current and electrical arc
- Automatic re-coupling following overcurrent and overvoltage events, if the fault does not persist
- Manual (after on-site inspection) re-coupling following leakage current and electric arc, events considered serious and life-threatening since they may imply electrocution or fire.
- Local display for status messages and data transmission to a concentrator for real-time monitoring via Web-based interface
- Built-in self-test (BIST) for checking system’s integrity at power-on and periodically during operation; local and remote notification of health status
- Protected against overvoltage of its own power supply

This device is thus designed not only as a replacement for the traditional circuit breakers, but an extension that offers enhanced protection and real-time notification features. In some cases, according to the deployment location’s specifics, due to the fact that the ELSA device is equipped with an execution element that has a maximum admissible current of 1kA, a classic short-circuit breaker with a larger short-circuit current (10kA) can be connected downstream of the device. This classical downstream breaker has therefore a higher decoupling current value than the ELSA breaker, which could increase the safety of the ELSA system in the case of a short-circuit where the current exceeds 1kA.

The protection device is based on a PIC 16F1829 microcontroller and can operate either in stand-alone mode, displaying its status on a local 7-segment display, or using a communication module to integrate it in an IoT network. The thresholds for load decoupling in case of overcurrent, overvoltage, leakage current and electric arc are programmable, and can be updated on-site by cable re-programming the microcontroller software (e.g. at user request). For future developments we envisage implementing remote, online update of these thresholds. This feature will be implemented with enhanced security (data encryption) to ensure the safety of the system.

**Fig 2:**
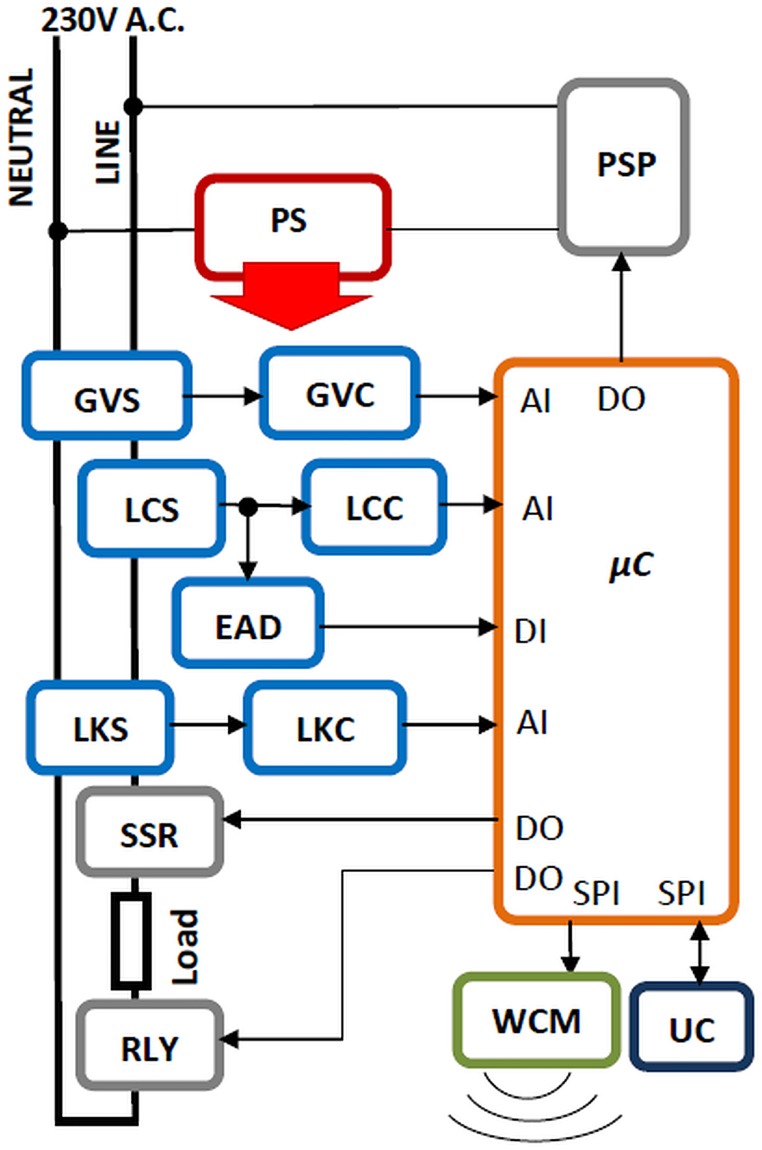
Internal architecture of the protection device.

An overview of the protection device’s architecture is depicted in Fig 2. A list and a short description for each of the internal modules is provided in Table 1.

**Table 1:** Internal modules description.

Module name	Description
*μ*C	PIC 16F1829 Microcontroller (where AI stands for Analog Input, DI –Digital Input, DO -Digital Output, SPI –Serial Peripheral Interface)
PS	Power Supply (this may be linear or switched)
PSP	Power Supply overvoltage Protection circuit
GVS	Grid Voltage Sensor
GVC	Grid Voltage Conditioning circuit
LCS	Load Current Hall Sensor
LCC	Load Current signal Conditioning circuit
EAD	Electric Arc Detection circuit
LKS	Leakage current Hall Sensor
LKC	Leakage current signal Conditioning Circuit
SSR	Switch for line connected to the load (Solid State Relay)
RLY	Switch for neutral connected to the load (Relay)
WCM	Wireless Communication Module (connected to CPU via SPI)
UC	User Control (A button for On (to connect the load) and Off (to disconnect the load), a button for Enabling and Disabling the electric Arc protection and a 7-segment display)

#### Central microcontroller unit and voltage/current sensors

The Microchip PIC 16F1829 microcontroller’s resources, 1KB RAM, 14KB Flash and an internal 32MHz oscillator provide appropriate performance for this application.

The GVC module receives the signal from the grid voltage sensor (GVS) following a low pass filtering for removing disturbing frequencies.

The input signal for the LCC module is provided by the Hall sensor output signal which underwent low pass filtering for removing the higher order harmonics.

In order for the EAD module to accurately detect the electric arc, a high pass filter selects the specific higher order harmonics encountered in the case of an electric arc and an analogic comparator compares the harmonics’ amplitude with a pre-defined threshold and activates the digital input DI.

In the LKC module, an operational amplifier is being fed with the input signal coming from a Hall sensor through which both phase and ground wires pass. The signal is proportional with the difference of the two currents in the phase and ground wires. Both signals generated by LCC module (proportional with the load current) and by the LKC module (proportional with the current difference between phase and ground) are applied to the analog inputs of the microcontroller.

#### Load decoupling circuit and power supply

For decoupling the load a static SSR module SSP1A125BD Schneider Solid state relay is used, having a 3-32V DC input, 24-300 V AC output, and 25A maximum current, and for neutral decoupling we have used a relay (RLY). Decoupling takes place first for the phase and then for the neutral, while recoupling occurs in the reverse order. The integrity of the SSR and the relay is being checked at device start-up and periodically during operation.

The PS power supply is a switching MEAN WELL DR-15-12 supply with a 12V DC output, 1.25A rated current and 85-264V AC supply voltage. The power supply is protected against overvoltage by the PSP (Power Supply Protection) circuit, consisting of a resistor connected in parallel with a triac. Under normal conditions, when the grid voltage is below a maximum value, the *μ*C activates a triac in order to short-circuit the resistor connected in series with the power supply. When the grid voltage exceed the maximum value, the *μ*C deactivates the triac, leading to the resistor being connect in series with the power supply, thus distributing the voltage between the resistor and the PS. When the grid voltage returns to nominal value, the *μ*C commands the re-activation of the triac.

Design and implementation precautions were taken in order to ensure that, among other potential disturbing factors, the opening and reclosing of the circuit breaker does not affect the operation of the microcontroller. Thus, the power supply has been specifically designed to provide a constant output voltage given any input voltage in the range 100-240V A.C. For voltages higher than 240V (up to 400V) the supply is provided with a protection that ensures a safe operation within parameters for an undefined period of time. Also, the microcontroller supports an input voltage between 1.8 and 5.5 V D.C. and it has an efficient low pass filter placed in the proximity of the supply pin.

Moreover, during the functional testing with all event types and a wide variety of RLC loads, the test stand continuously generated all types of fault events and measured the decoupling/recoupling timings. During these tests and sequences of coupling/decoupling the breaker, the microcontroller operated unaffected by any disturbances.

#### Modular concept of the ELSA device

The WCM (Wireless Communication Module) is connected to the microcontroller via SPI and can be of different types, according to the client’s / deployment location’s requirements. For the household deployment of ELSA, we envisaged a 3G WCM transmission module, in which case the communication with the data concentrator for event signaling is performed using the mobile phone network. For the intelligent lighting project under implementation with the local municipality, we used a LoRa (Long Range Wide-area network) WCM module with a maximum transmission range of about 10km in order to ensure integration with the existing LoRa-based network deployed throughout the city. In the case of deploying the ELSA device in households located in large apartment buildings, we envisaged as the most favorable solution an RF WCM module. Thus, the laboratory tests were performed using RFM12B, a transceiver with FM modulation and a typical transmission range of about 100m, meeting the field requirements in this deployment scenario, for sending data from each ELSA device to a local concentrator in each apartment building. In this scenario, securing the data was performed using 16-bit encryption, and the local concentrator sends the data forward to the global concentrator via Ethernet. The ELSA device was also programmed in order to periodically check the health status of the connection with the local concentrator by sending a health-check message and waiting for the response—in case of a communication failure this is signaled as an error code on the local display. Currently, we are looking (together with the local municipality) into ensuring a higher grade of IoT-compatibility regarding the communication part of ELSA according to the international standards promoted by the Wi-SUN alliance (Wireless Interoperable Smart Utility Networks).

The ELSA protection device has a modular concept, i.e. can be used in various configurations. The device can be configured using the basic modules that ensure either only phase or both phase and neutral decoupling, to which a transmission module can be added. Several configurations as identified by potential users were laboratory tested:

- Basic protection device without communication module
- Basic protection device with just phase decoupling
- Protection device with Ethernet communication module (household use)
- Protection device with 3G communication module (household use)
- Protection device with LoRa communication module (for integration in the smart city network)
- Portection device with RF module (for users living in apartment buildings)

Other communication modules can be accomodated by the device, e.g. WiFi or PLC (Power Line Communications), but these configurations have not been tested yet.

### Embedded software

**Fig 3:**
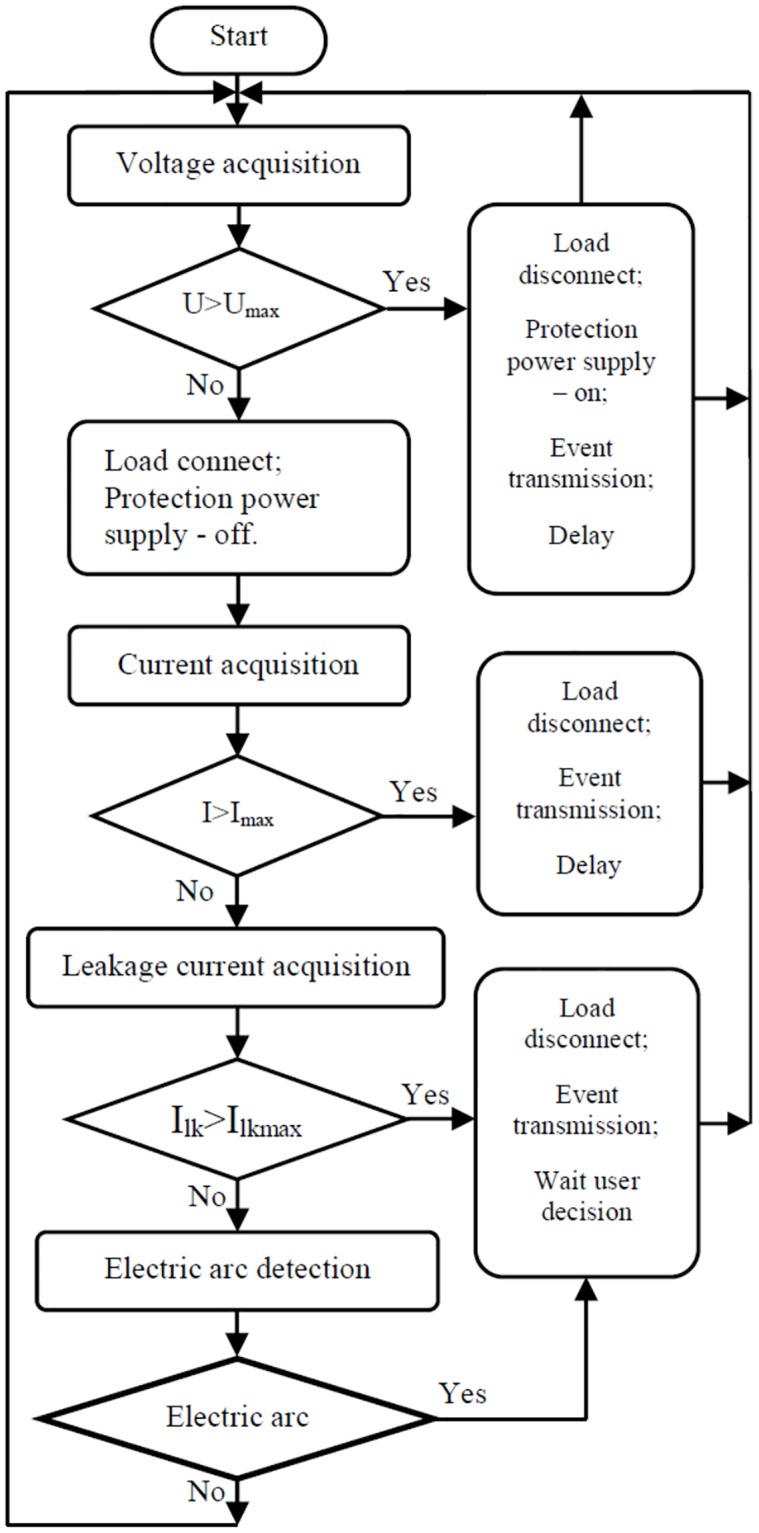
Embedded software control flow diagram.

Fig 3 illustrates the control flow diagram for the embedded software running on the PIC 16F1829 microcontroller.

The diagram shows the sequential measurements and checks for grid voltage, load current, leakage current and the presence of an electric arc. In each of these measurement stages any exceeding of the maximum threshold (previously programmed) will lead to the decoupling of the electrical load. In the event of overcurrent or overvoltage, the system attempts to automatically re-couple the load after a specific programmed interval has elapsed. In case the fault persists, the load remains de-coupled; a maximum of three automatic re-coupling attempts are performed.

Following a decoupling caused by an electric arc or leakage current, re-coupling can be made only after a manual user confirmation (performed by pushing a button on the device—in the User Control module) given the potential dangerous circumstances involved in the case of such events.

While the diagram shown in Fig 3 displays a sequential flow of the operations, the practical implementation of the software is based on the interrupt system of the microcontroller. Thus, the interrupts are generated by both external (arc electric detection and front panel events) and internal sources (microcontroller timers dedicated to the generation of pre-programmed time intervals).

The detection of an electric arc generates an interruption that enables Timer 1 to determine the duration of that electric arc. The event will cause the load to be decoupled if the persistence determined is greater than a predefined threshold. Timer2 is used to generate the sample rate (period of 250us). Each Timer2 interrupt triggers a routine that reads analog values of grid voltage, load current and leakage current. If any of these values exceeds the limit, the load decoupling is performed (both switches turn off). The healthy state of the switches is determined periodically following self-tests generated by interrupts triggered by Timer3.

### Communication interface and data concentrator

The communication infrastructure fosters data transmission from each protection device towards a Web server that centralizes it and provides an online Web-based interface for remote monitoring and control.

This infrastructure has been designed and implemented using IoT-compatible technologies and design paradigms, including optimized Internet protocols (based on HTTP requests) and communication standards. The communication system represents an extended development of a previous implementation described in [35].

In the context of the modular concept of the ELSA protection device, several communication configurations have been tested. We have focused our efforts on two in particular: the RF communication with local concentrator for deployment in apartment buildings (described below) and the integration in the city’s smart network (using LoRa).

**Fig 4:**
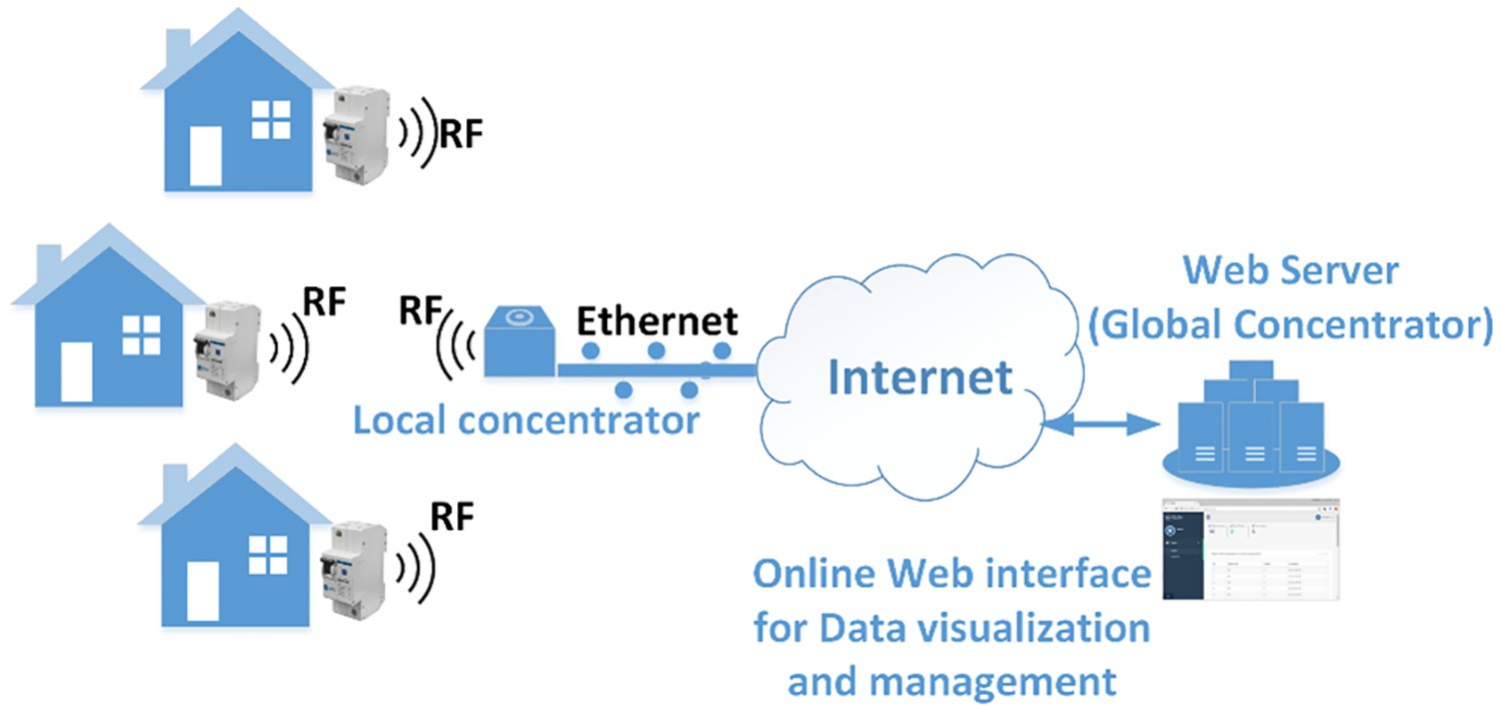
Overview of the communication system architecture.

The communication system for the household apartment-building scenario comprises embedded networking (RF) capabilities in each protection device, a (local, in the field) data concentrator, a Web server (global concentrator) and a Web-based interface for monitoring and control of the entire system. Fig 4 displays an overview of the communication system’s architecture.

Each protection device embeds an RF module connected to the microcontroller that is used to transmit the status and events detected to the local concentrator, which is also a microcontroller device integrating an RF module and Ethernet shields. The local concentrator receives RF broadcasts from the protection devices in range, confirms the receive by sending back an acknowledgement, processes their input, wraps it up in JSON format and sends it as a HTTP request to an URL representing an API (Application Programming Interface) point of the global concentrator (which is actually a Web server). In order to enhance the reliability of the system, the local concentrator periodically sends “status check” messages to each of the protection devices in its range and expects a health status report in order to verify their functionality from time to time.

Thus, the communication infrastructure based on a local concentrator is very similar to the one used in advanced metering infrastructures (AMI), where a local concentrator is also used for collecting electricity usage data from residential electricity meters [36], using Wi-Fi or PLC (power line communication technologies) [37].

The global concentrator, a node.js Web Server implemented and deployed on a Linux machine in our department (running the http://etc.unitbv.ro domain), provides an API used for communicating with the local concentrator devices by HTTP GET-POST requests encapsulating data as JSON strings in the requests’ body. Data storage and management is accomplished using a PostgreSQL database interfaced using specific node.js modules. In addition to adding each event to the map, an e-mail and SMS message (Short Message Service) are being sent to designated contacts for real-time notification.

Data communication between the local concentrators and the Web server for transmitting information received from the protection devices has been implemented based on GET or POST requests made from the local concentrators to the URL http://etc.unitbv.ro/elsa/api/v1/reports with the data encapsulated in the request’s body as a JSON string.

**Fig 5:**
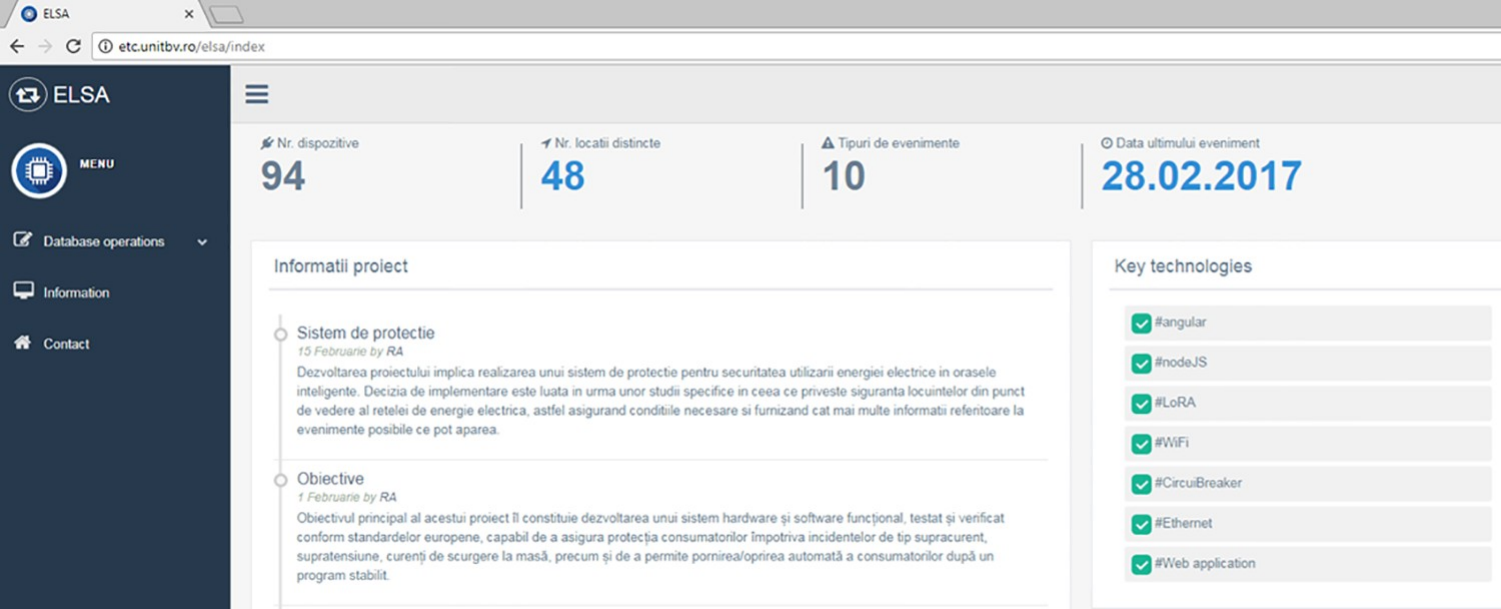
Web interface for remote monitoring of the protection devices—Homepage view.

The Web-based interface (Fig 5) has been implemented using JQuery, Bootstrap, CSS and HTML and displays information about the operational protection devices (the recorded events in the database and their current status). This approach is a renowned practice in this field in order to make interaction with the protection devices easier by using a Web application (like the WebIoT app [38]). The Web page is easily scalable according to the displaying device specifics, with enhanced support for Mobile browsers included. The interface is accessible on a public URL (http://etc.unitbv.ro/elsa), access being regulated based on user authentication and privileges.

**Fig 6:**
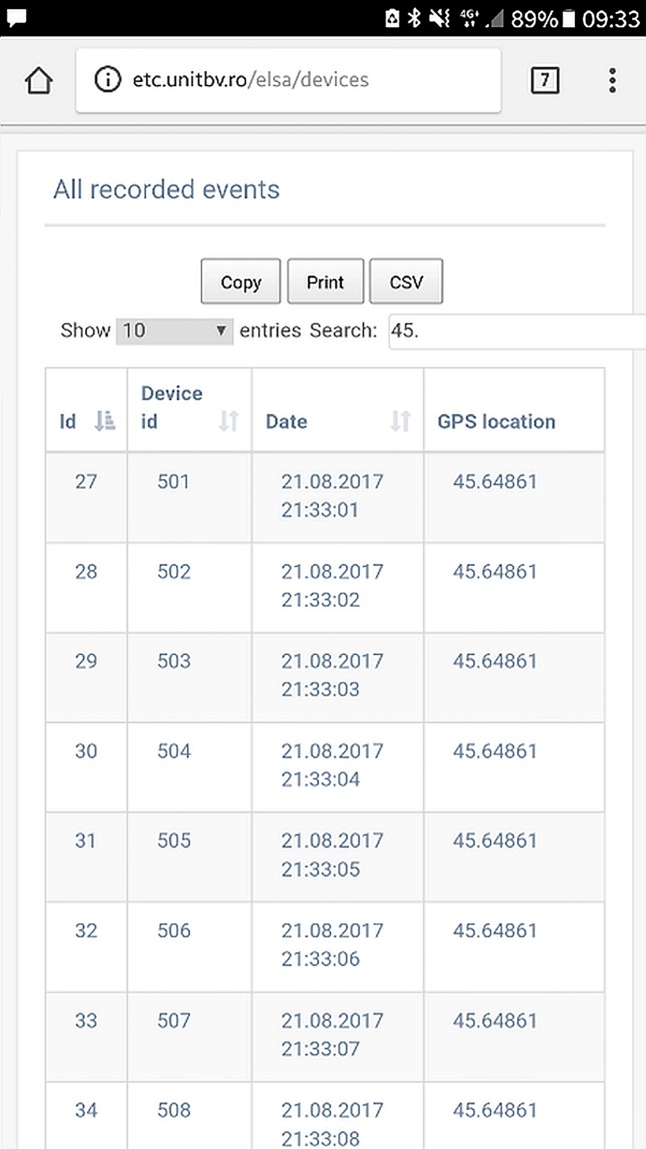
Screenshot from a mobile device showing the recorded events by the devices in a table format.

The Web interface provides both a table view (as shown in Fig 6) and a geo-referenced visualization of the protection devices and recorded events using the Google Maps JavaScript API. A Google Map is instantiated and populated with markers corresponding to each recorded event from the database. A mapping has been made to associate each event type a different marker color for better visualization. Extensive details about each event are displayed when the corresponding marker is clicked on the map.

In designing the protection device we have envisaged integrating the concepts and paradigms of the Internet of Things [39] in order to provide advanced, remote monitoring of the events and real-time notifications. Such an approach fostering the synergy between the arising IoT and traditional electrical protection system has the benefits of providing improved scalability, security and interoperability of the entire system, and pertains to a newly emerging concept of the Internet of Energy (IoE) [40], thus meeting the requirements necessary for integrating our protection system in Smart City IoT-based infrastructure in Brasov.

To summarize, our approach for transcending a traditional electrical protection system to one that is “IoT-ready” brings the following important features:

- Easy and uniform interaction with other communication and data systems due to the effectiveness of IP-based networking [41].
- Low-power overall consumption by integrating low-power microcontrollers and communication shields. (Thus enforcing the current trends in IoT communication devices [42]).
- Simplified communication architecture based on HTTP requests, consequently reducing the complexity of the hardware/software implementation.
- Interoperability—achieved by using IP based communication, HTTP requests and JSON-structured messages—that allows a seamless integration with other systems.
- Scalability—the architecture of the entire communication system allows connecting a variable number of devices “on the fly”.
- Easy data exchange with other entities (e.g. utility companies, emergency services).

## Results and discussion

### Load decoupling and recoupling simulations

**Fig 7:**
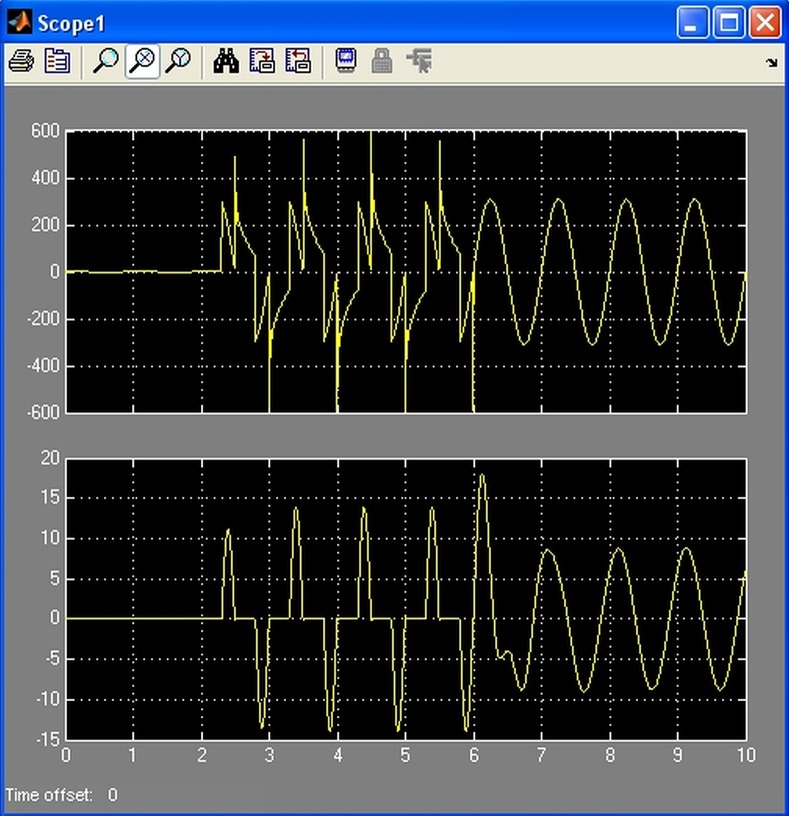
Simulation showing load coupling with variable triac firing angle. Voltage (up) and current (down) load variations for a RLC load.

Simulink simulations were performed prior to device implementation, as shown by Fig 7. These simulations implied the development of a simulation model of the execution element (triac and associated components) and the study of the waveforms during connection/disconnection for different combinations of R, L and C. The simulation also considered slow start recoupling by commanding a variable triac firing angle. According to the simulation, the currents’ waveform for some loads was altered to such an extent, therefore these load scenarios have not been implemented in practice.

### Functionality and reliability testing in laboratory

The ELSA protection device underwent functional testing with R, L, and C loads in different combinations, measuring the decoupling speed for various faults and also validating the data communication infrastructure. In order to accurately determine the reliability of the device, a dedicated test stand has been designed and developed that continuously generates all types of fault events and measures the decoupling/recoupling timings. This stand also supports accelerated reliability testing, including temperature and humidity variation, according to standard EN 62059-31-1.

**Fig 8:**
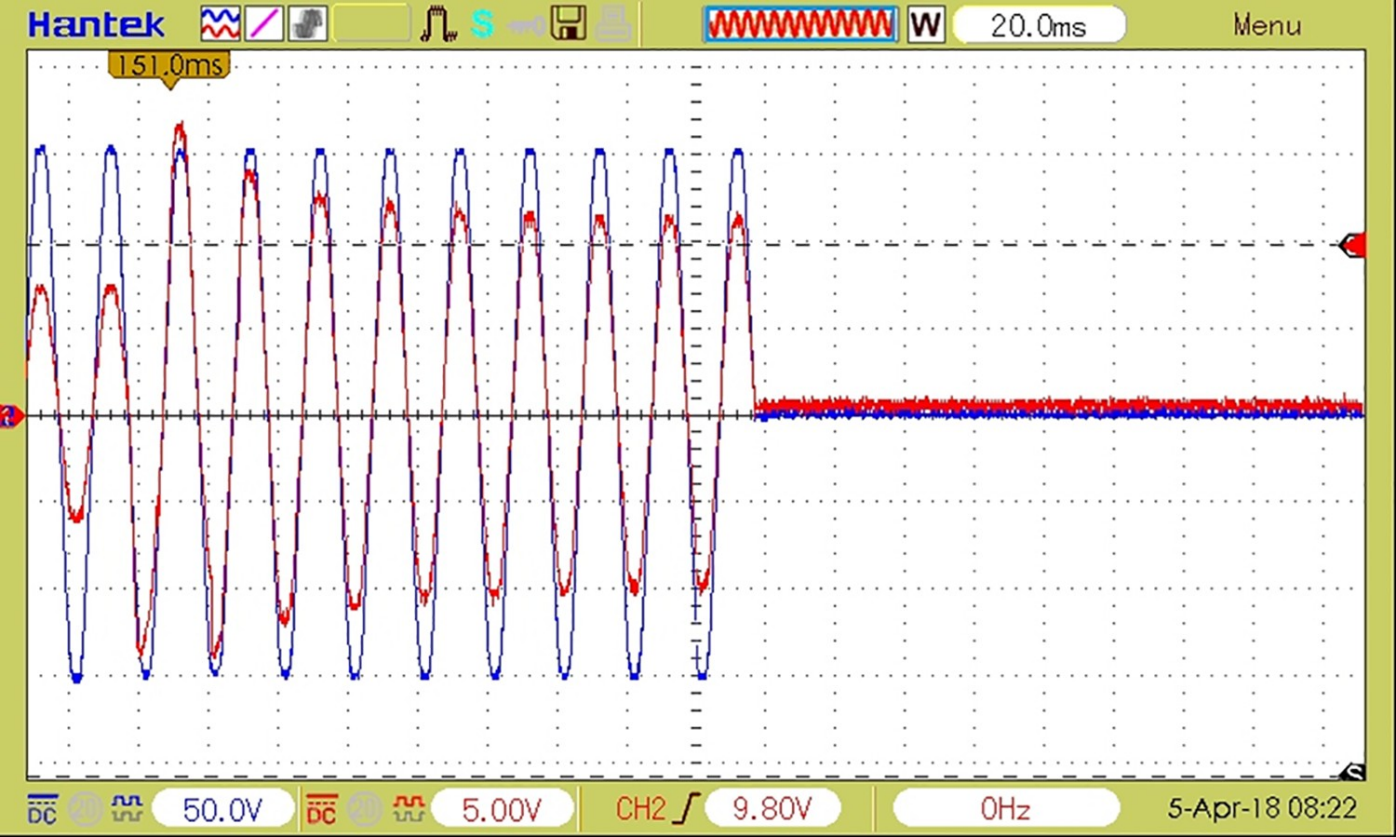
The waveforms of load current and voltage at coupling an additional R load causing the decoupling in 10 periods—200ms (in the event of a 20% overload).

Fig 8 shows the waveforms corresponding to load voltage and current during a coupling followed by a decoupling. In this figure, the load voltage is represented by the blue sinusoidal signal, and the load current by the red sinusoidal signal for an R load for a scenario in which a 20% higher overload than the admissible threshold is detected. In this case, event detection and decoupling timings are similar to those of a traditional circuit breaker. The waveforms in the case of decoupling and recoupling are shown for the event of overload detection, but are similar for any other fault event (electric arc, leakage current or overvoltage), only the decoupling time interval varies.

**Fig 9:**
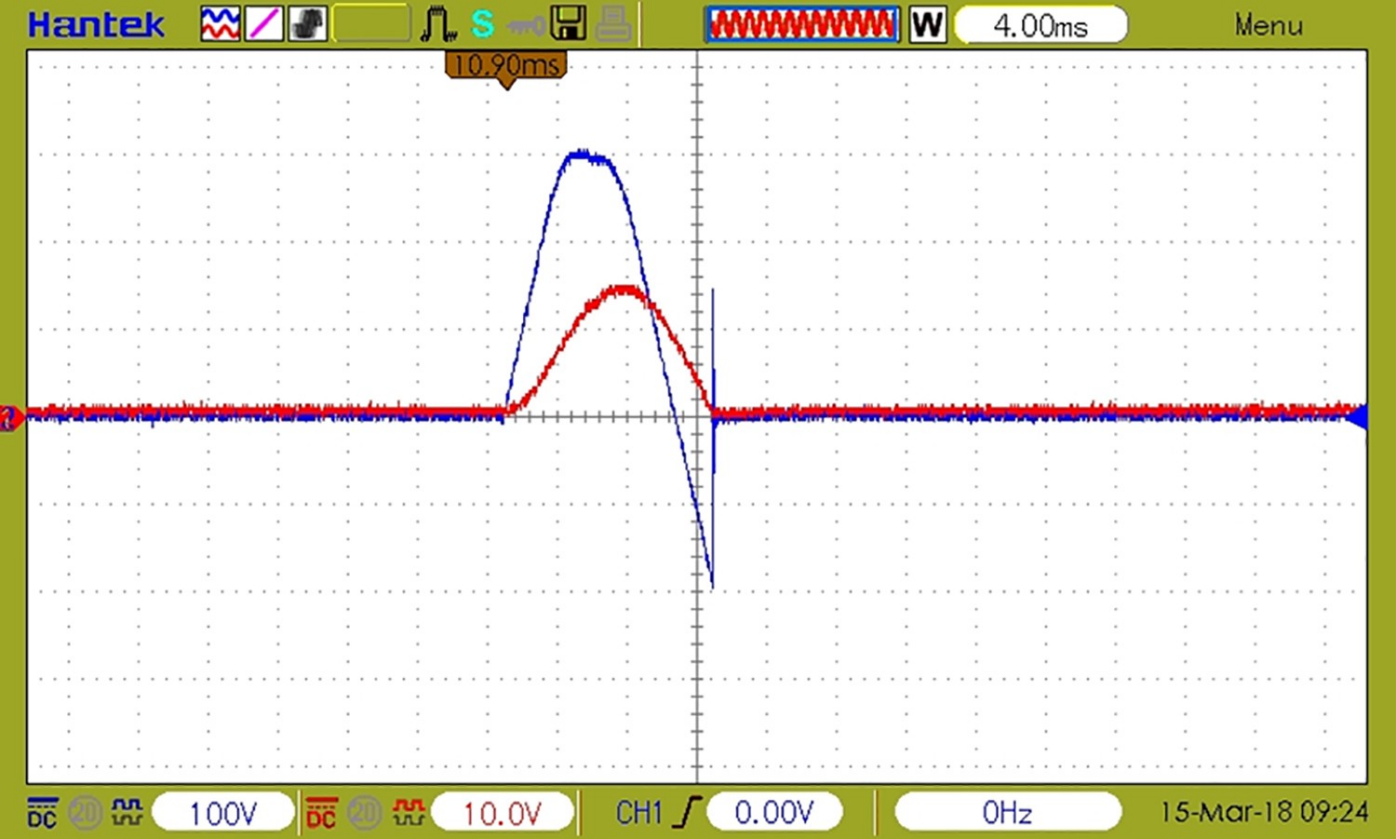
The waveforms of load current and voltage at coupling followed by decoupling of RL load. Decoupling time 10ms, overload 50%.

Fig 9 depicts a coupling with a duration of 1 semi-period for a RL load and the sudden voltage variation at decoupling, during the current’s zero crossing. The waveforms correspond to an overload event with a 50% higher load than the admissible threshold.

The figure also shows that both load coupling and load decoupling are performed by the device at the current’s zero crossing, which ensures minimum perturbations being generated in the electrical grid. The analysis of current samples and detecting the faulty event takes one period of the grid’s voltage.

**Fig 10:**
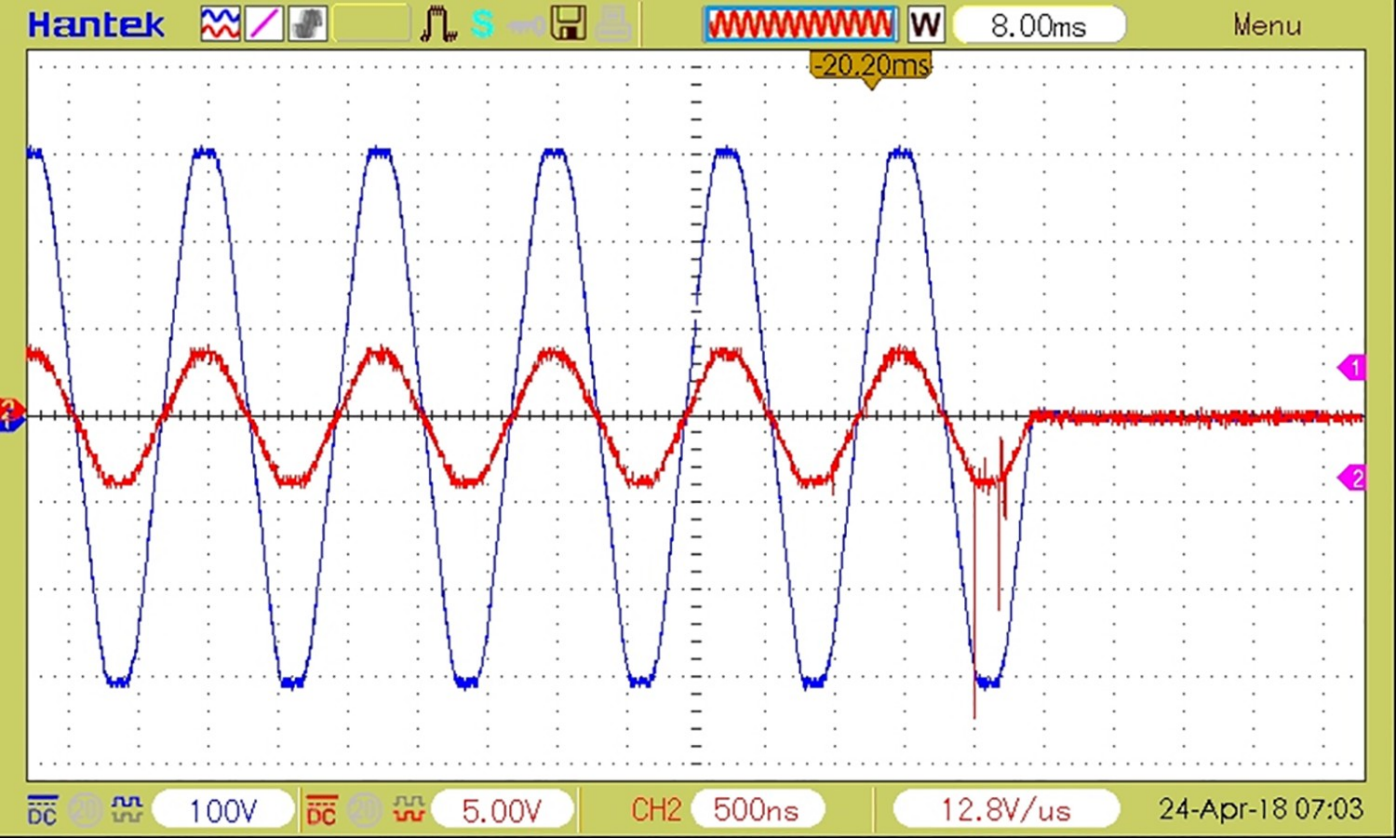
The waveforms of load current and voltage at decoupling caused by an electric arc detection on a resistive load.

A decoupling event caused by an electric arc detection is depicted in Fig 10, where the current variations caused by the electric arc are visible.

An analysis was also performed in order to proper handle various failure scenarios caused by malfunctions of the ELSA device. In the case of decoupling/recoupling faults a specific error message is shown on the local display together with an error notification message sent to the user via SMS. If the malfunction involves critical system components (e.g. the microcontroller or power supply), the device still offers basic protection against short-circuit until the defects have been remedied. In this case, remote fault detection is performed by the server upon not receiving the status message from the device which is supposed to be sent one each 24 hours.

### Precompliance EMC testing

The protection device has been subject to pre-compliance tests according to European standards in Electromagnetic Compatibility [43].

A summary of the performed EMC tests is shown in Table 2.

**Table 2:** Electromagnetic compatibility (EMC) tests performed on the protection device.

Standard name	Description
EN 61000-3-2	EMC—Part 3-2: Limits—Limits for harmonic current emissions (equipment input current ≤ 16 A per phase)
EN 61000-4-11	EMC—Part 4-11: Testing and measurement techniques—Voltage dips, short interruptions and voltage variations immunity tests.
EN 61000-4-13	EMC—Part 4-13: Testing and measurement techniques—Harmonics and interharmonics, low frequency immunity tests: “Flat curve”, “Over Swing”, “Sweep in frequency”, “The individual Harmonics”, “Interharmonics” and “Meister Curve Test”
EN 61000-4-14	EMC—Part 4-14: Testing and measurement techniques—Voltage fluctuation immunity test for equipment with input current not exceeding 16 A per phase.
EN 61000-4-28	EMC—Part 4-28: Testing and measurement techniques—Variation of power frequency, immunity test.

The tests were performed at the Research and Development Institute of Transilvania University of Brasov, Romania, which accommodates an advanced system for testing electrical and electronic equipment under electromagnetic disturbances which provides instruments for testing electromagnetic immunity and interference in a frequency spectrum up to 18GHz. This integrated testing system includes an ensemble of shielded rooms and an EM Test NetWave 30 equipment dedicated to measuring electromagnetic disturbances generated in the power grid and electromagnetic immunity (voltage/frequency variations, harmonics, etc.).

The protection device has been implemented in two prototypes having two different configurations of the power supply: one with a linear and the second with a switching supply.

Two types of tests were performed: tests for grid perturbations generated as high harmonics and immunity tests in which the device’s functionality was verified under perturbations generated using the EM equipment. The first type of tests were performed by measuring the value of the high harmonics according to the EN 61000-3-2 standard. The second type of tests aimed at testing the device’s immunity to the following:

- Voltage dips, short interruptions and voltage variations, according to EN-61000-3-2
- Higher harmonics originating from various malformations of the sinusoidal signal of the power grid, according to EN 61000-4-13
- Voltage fluctuation, according to EN 61000-4-14
- Variation of power frequency, according to EN 61000-4-28

Both prototypes, regardless of power supply type, passed all tests. Nevertheless, higher order current harmonics generated in the grid are higher in the case of switching power supply.

**Fig 11:**
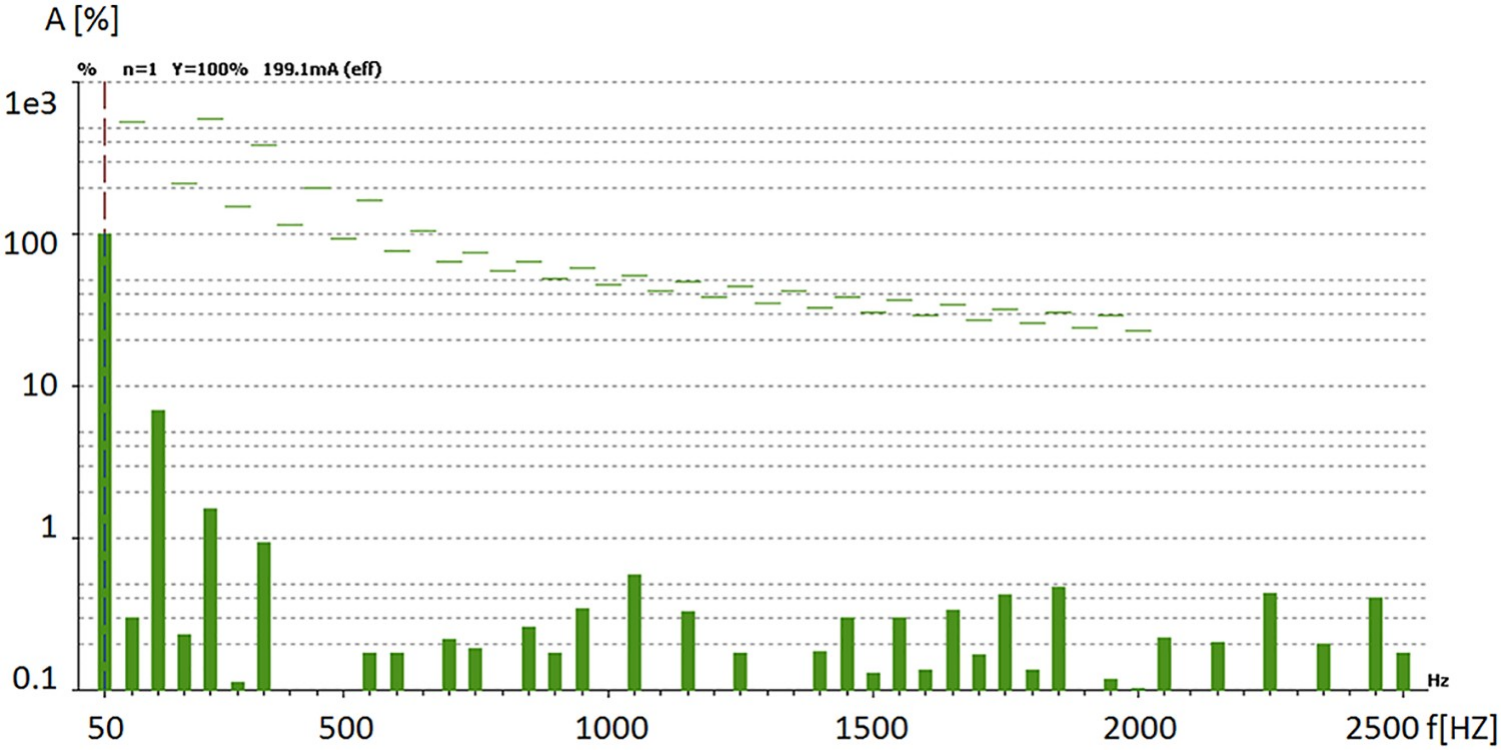
Higher order harmonics generated by the linear power supply.

**Fig 12:**
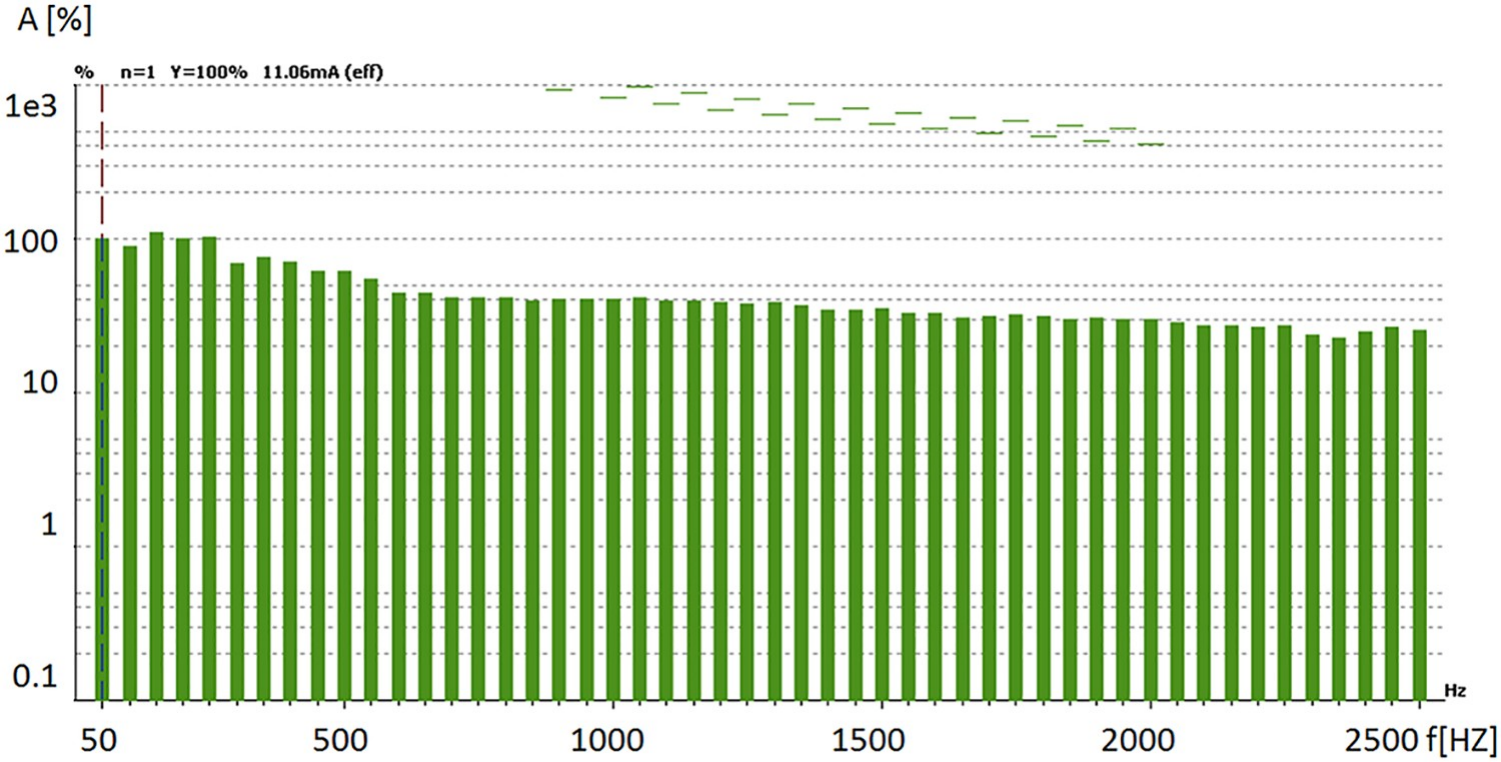
Higher order harmonics generated by the switching power supply.

Fig 11 presents a diagram showing the amplitude of the harmonics generated in the power grid as percent of the fundamental frequency, together with the maximum admissible values (dotted lines) for the linear power supply, while Fig 12 shows the same harmonics but for the switching power supply.

**Fig 13:**
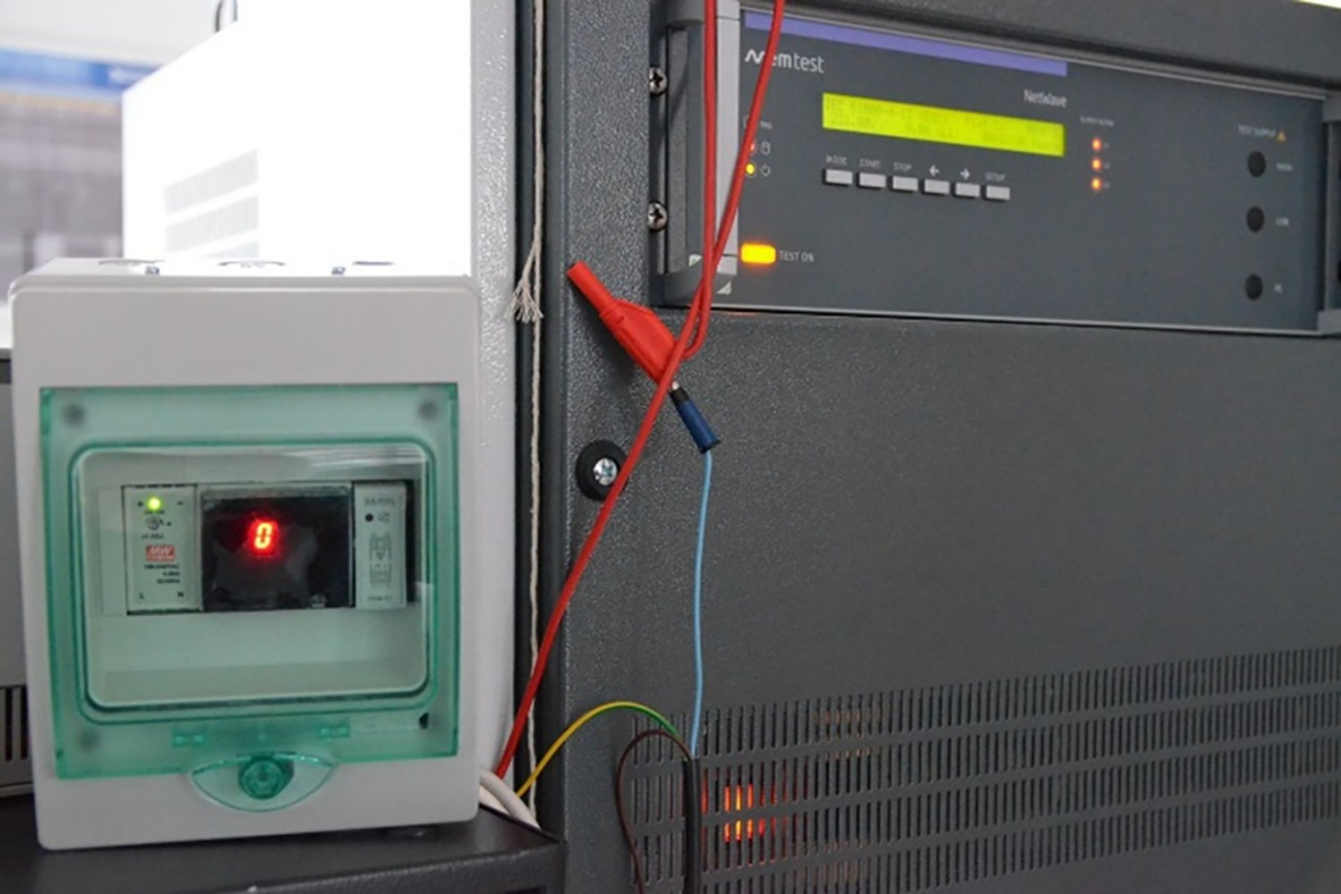
ELSA device during EMC tests.

The protection device undergoing the afore-mentioned EMC tests connected to the EM Test equipment is depicted in Fig 13.

## Conclusion and future work

This paper described the design, implementation and functional validation of an advanced power-system protection device with IoT-based support for integration in smart environments like Smart Homes or Smart Cities.

The protection device ensures the safety of electrical consumers connected to the public power supply grid by disconnecting the electrical supply in the event of several faults: overvoltage, overcurrent, leakage current and electrical arc.

The proposed system transcends the traditional functionalities of a classic circuit breaker not only by providing protection against additional faults but also by “becoming smart” in the sense that each protection device communicates using a concentrator-type architecture with a Web server for reporting the recorded events. The system also provides a Web-based interface for monitoring the network of operating devices and also real-time event notification through e-mail and SMS messages.

The protection device underwent successful functional testing with R, L, and C loads in different combinations, measuring the decoupling speed for various faults and was also subject to pre-compliance tests according to European standards in Electromagnetic Compatibility, passing all tests. Also, we have implemented a test bench for assessing the reliability of the device under stressful conditions (generating sequential anomalies—overcurrent, overvoltage, leakage currents and electric arcs) and monitoring coupling/decoupling at various temperatures. Extended reliability testing is currently underway using this test stand. The current development stage of the ELSA device is TRL 4 (Technology Readiness Level), i.e. the device has been validated in laboratory conditions.

Future developments will include integrating the protection device in the smart city infrastructure of Brasov city, more specifically the smart public lighting system with LoRa (Long Range Wide-area network) communication support. Also, we envisage replacing the coupling/decoupling component—which is a SSR (Solid State Relay) and thus has a maximum admissible current of 1kA—with an element capable of supporting higher currents for enhanced protection against short circuits that may generate higher current values th at SSR can handle.

Last but not least, prospective tests envisage the integration of the ELSA device in power grids with distributed energy generators for identifying and addressing potential technical issues. Hence, we are considering as an important research direction extending the functionality of the ELSA device for ensuring the protection of consumers connected to DC micro-grids. In the initial design stage we aimed at having a high degree of flexibility for the device, based on a modular concept, such that a potential user could choose the desired type(s) of protection, the maximum admissible values for the parameters of the voltage/current and the desired data transmission. As such, developing different decoupling modules for both AC and DC grids is an ambitious goal that we are currently pursuing.
